# The Influence of COVID-19 on Medical Student Resource Preferences

**DOI:** 10.7759/cureus.28593

**Published:** 2022-08-30

**Authors:** Phillip M Johansen, Lindsay Celentano, Adam T Wyatt

**Affiliations:** 1 Neurological Surgery, Florida Atlantic University Charles E. Schmidt College of Medicine, Boca Raton, USA; 2 Medical Education, Florida Atlantic University Charles E. Schmidt College of Medicine, Boca Raton, USA; 3 Integrated Medical Sciences, Florida Atlantic University Charles E. Schmidt College of Medicine, Boca Raton, USA

**Keywords:** self-directed learning, medical education, instructional methods, educational resources, covid-19

## Abstract

Introduction

Over the past decade, pre-clerkship medical education has shifted from solely relying on didactic lectures to implementing more group learning and clinical experience to promote individualized, self-directed, and patient-centered education. COVID-19 required medical schools to examine their curricula and determine which portions were adaptable to virtual learning. This study compared first-year medical students’ (MS1) perceptions of an online curriculum, focusing on the students’ preferred resources before and after the transition to virtual courses.

Materials and methods

At one community-based allopathic medical school, a decision was made to move the entire pre-clerkship curriculum to a virtual format in the setting of the pandemic. An end-of-course survey evaluation was distributed via email to 64 first-year medical students at a community-based allopathic medical school. The participants were asked numerous questions about their overall perceptions of each course, including questions about the usefulness of lectures, small group activities, course administration, and faculty communication. Quantitative and qualitative data were collected during the standard program evaluation process for the two courses, and a third survey that focused on learning resources asked questions regarding virtual learning.

Results

Of the students, 29.7% reported being disappointed with the virtual curriculum, while the other 70.3% reported an unchanged or improved overall medical education. Regarding resource preferences, 56.5% of the students viewed most (76%-100%) course lectures, while 35.5% of the students viewed less than half of the course lectures. In contrast, 75.8% of the students said the majority (>50%) of their learning comes from outside resources. Furthermore, 31% reported that they are satisfied with the resources provided by the school, while 42% reported that they would like the school to provide additional resources. With that being said, 61% reported using more outside resources with the onset of a virtual curriculum, while 34% reported no change in outside resource use. Only 2% reported using fewer outside resources.

Conclusion

This study found that pre-clerkship medical students preferred some aspects of the in-person setting, such as social interaction and clinical exposure that is lacking in the virtual setting. However, students preferred many aspects of the virtual setting, such as having more independent study time and a more efficient learning process. Overall, before and after the transition, students were less satisfied with traditional curricular resources and more likely to choose external, board-specific resources with hopes of building strong residency applications, and these preferences were heightened in the online format.

## Introduction

Over the past decade, pre-clerkship medical education has shifted from solely relying on didactic lectures to implementing more group learning and clinical experience to promote individualized, self-directed, and patient-centered education [1-3]. Student assessment has also moved accordingly, with pass/fail, competency-based programs beginning to take the place of traditional letter-grade systems [4,5]. Such pass/fail assessments were largely designed to reduce perceived competition between medical students and foster a cooperative environment, which mimics the real-world conditions that students will encounter in the healthcare field [6]. This pass/fail system has improved the general well-being of medical students but has also increased the importance of standardized tests for students aiming for competitive residency positions [7].

While medical schools are working to keep up with the changing trends in education, the historical importance of United States Medical Licensing Examination (USMLE) Step 1 in the residency selection process resulted in students substituting traditional didactic lectures for board-specific test preparation resources, such as First Aid, Boards & Beyond, or Sketchy Medical, more than ever before in an attempt to raise their scores and maximize their efficiency [8]. Results from the Association of American Medical Colleges (AAMC) Year 2 Questionnaire (Y2Q) reveal that 70.1% of students (up from 47.7% in 2015) utilize online medical education videos (e.g., YouTube, Boards & Beyond, and Sketchy Medical) at least once a week or more, and 77.3% (down from 85.7% in 2015) report using other online medical education resources (e.g., Wikipedia and UpToDate) at least once a week or more. In comparison, 39.9% of students (down from 52.3% in 2015) attend in-person lectures at their school “often” or “most of the time,” while 54% (up from 52.5% in 2015) watch their school’s virtual lectures “often” or “most of the time” [9]. These trends reveal that more students use outside resources than their schools’ resources, and even those who do report using their schools’ resources feel the need to look elsewhere to supplement their undergraduate medical education (UME).

Because of this documented shift to resources not provided by the institution and with COVID-19 forcing medical schools to adopt new, virtual curricula, students are facing more barriers to traditional in-person UME than ever before such as ineffective teaching and learning strategies, poor communication, and lack of collaborative learning experiences [10,11]. These obstacles to traditional UME have likely made the transition to online study aids even more prevalent. Further, with some schools considering making hybrid learning models permanent, student selection of outside learning resources, such as board-specific programs, will likely exacerbate existing issues. This study attempts to better understand medical students’ resource preferences while examining how COVID-19 and the associated virtual curriculum have changed the resources that medical students are using.

## Materials and methods

Participants

In this observational study, an end-of-course survey evaluation was distributed via email to all 64 first-year medical students at a community-based allopathic medical school. All students participated in the in-person course immediately before and the online course immediately after COVID-19 forced schools to transition to a virtual curriculum. The participants voluntarily completed all surveys. These surveys were completely anonymous, and no identifying data was contained therein. This study was waived by the Institutional Review Board (IRB) at Florida Atlantic University.

Data collection

Course evaluation surveys were completed immediately following each course. The survey regarding resource use was completed immediately following the virtual course. The participants were asked numerous questions about their overall perceptions of each course, including questions about the usefulness of lectures, small group activities, course administration, and faculty communication. Additionally, the students were asked about the amount of independent study time they had during each course, as well as the resources they utilized, the amount of time spent utilizing each resource, their satisfaction with the resources available, and how these preferences differed between the courses.

Statistical analysis

We utilized a mixed method approach to analyze the perspectives of first-year medical students during two systems-based courses in which one course was in-person and the other virtual, due to COVID-19. Quantitative and qualitative data were collected during the standard program evaluation process for the two courses, and a third survey that focused on learning resources asked questions regarding virtual learning. Quantitative data were analyzed using standard analysis of variance procedures (SPSS version 28.0, IBM Corp., Chicago, IL, USA). Post hoc tests were also conducted to determine the exact differences seen in the study population. Qualitative data were analyzed using content analysis (Taguette, Open Source Collective, Delaware, USA) [12].

## Results

Sixty-four first-year medical students completed Likert-like scale surveys after organ system modules, one of which was in-person (Neuroscience and Behavior) and one of which was virtual (Gastrointestinal System). Students felt similarly about the courses in terms of content delivery and helpfulness of course activities, with the biggest difference being that the communication between course directors and students was more difficult in the virtual setting. However, one of the most notable findings was that the amount of independent study time was greater with online classes (mean (M) = 4.47, standard deviation (SD) = 0.71) than with in-person classes (M = 3.77, SD = 1.24, t = -3.92, p < 0.01). This is significant when taken in context with the finding that students often remarked this increase in free time was largely due to the use of concise, online test preparation resources instead of online lectures.

**Figure 1:**
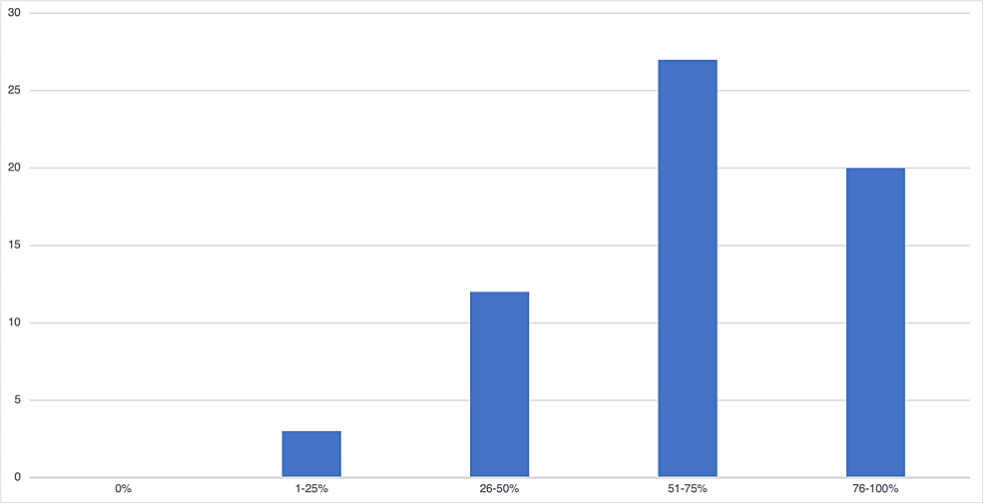
Percentage of learning coming from outside resources Students were asked, “What percentage of your learning comes from outside resources?” The responses were based on a Likert scale ranging from 1 to 5, with 1 corresponding to 0% of learning coming from outside resources, 2 corresponding to 1%-25% of learning coming from outside resources, etc. The graph shows the number of students who fall in each category.

When asked about the use of online lectures, students often reported watching lectures at an accelerated speed, and answers were mixed on whether these school lectures were a “primary lecture tool” or a “review tool.” For example, one student reported that online courses allowed them to “watch (lectures) on (their) own schedule, pause to take notes, rewind … or speed up the (lectures),” while another wrote, “I find that the lectures confuse me more than more concise mediums, like Boards & Beyond … I want to watch lectures, but I feel like I’m wasting my time.” Such statements are consistent with our data, which showed that only 56.5% of the students viewed most (76%-100%) course lectures, while 35.5% of the students viewed less than half of the course lectures. In contrast, 75.8% of the students said the majority (>50%) of their learning comes from outside resources (Figure 1), which is similar to findings reported in the AAMC Y2Q survey [9]. When asked what these outside resources were, Boards & Beyond, Pathoma, Sketchy Medical, and Anki were the most commonly reported.

When asked about how they felt their medical education was impacted, 29.7% of the students reported being disappointed, but the other 70.3% were generally pleased with the online curriculum because they had more time to study. For example, one student commented, “fewer in-person activities and no travel time has lent me more time to study course material,” and another stated, “my learning has become much more independent and self-driven.” However, on the other end, one student lamented, “I think this experience has taken away my ability to form more meaningful relationships with faculty and community affiliates, (and) I have lost many opportunities for clinical education … I think I would have learned the course content better if we were in person.”

**Figure 2:**
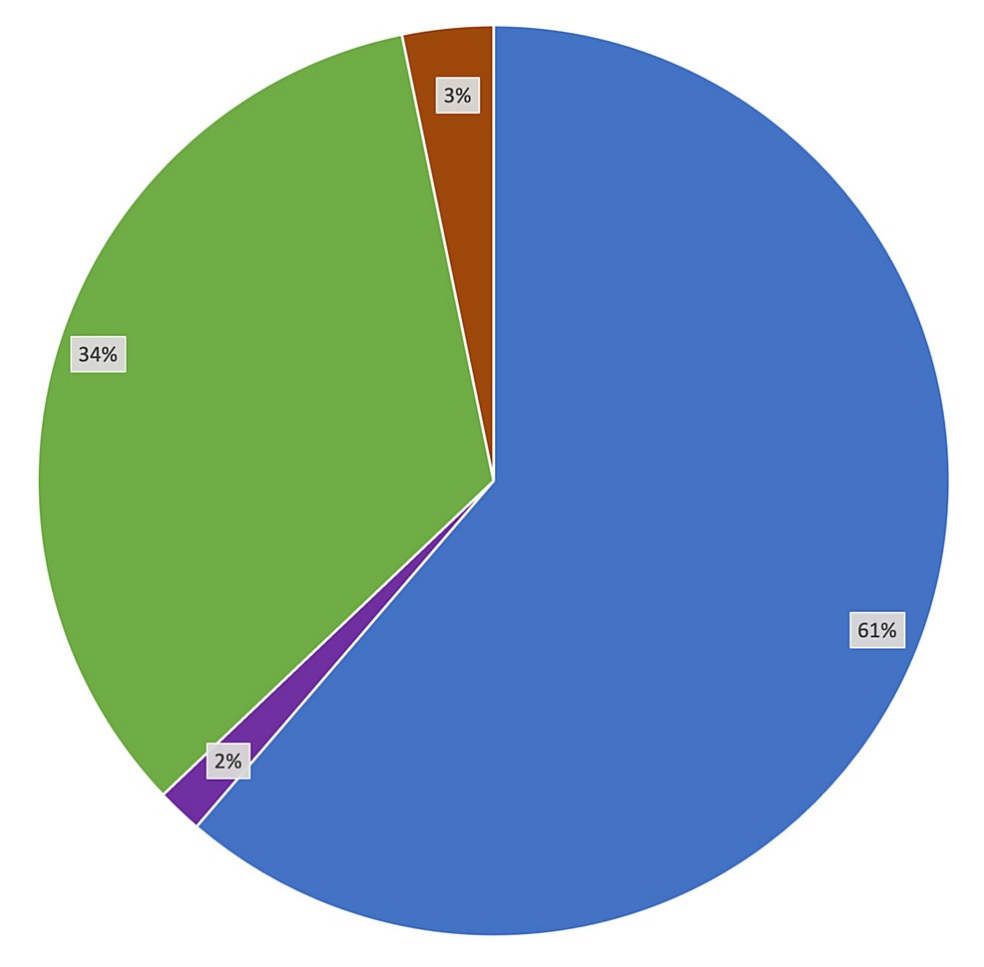
Change in outside resource use after the onset of a virtual curriculum Students were asked, “How has the number of hours you spend per week using outside resources changed since the onset of COVID-19?” Blue: increase; purple: decrease; green: no change; red: no response

In terms of provided resources, 31% of the students reported that they are satisfied with the resources provided by the school, while 42% reported that they would like the school to provide additional resources. With that being said, 61% of the students reported using more outside resources with the onset of a virtual curriculum, while 34% reported no change in outside resources. Only one (2%) student reported using fewer outside resources (Figure 2). Additionally, students reported that anatomy laboratory (M = 4.06, SD = 1.01) was more useful than the online anatomy applications provided by the school (M = 3.66, SD = 1.20, t = 2.31, p < 0.05) and that the required texts were not so useful (M = 2.24, SD = 1.16).

## Discussion

**Figure 3:**
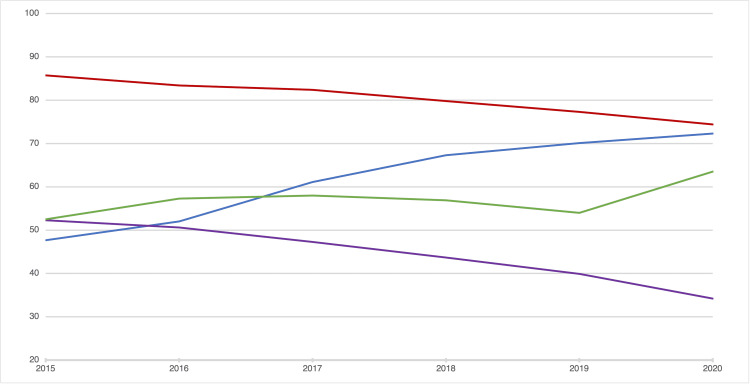
AAMC Y2Q survey 2015-2020: percent student utilization by resource type Blue: use of online videos for medical education information ≥1× per week; red: use of other online content for medical education information ≥1× per week; purple: regular use of in-person pre-clerkship courses/lectures at your medical school; green: regular use of virtual pre-clerkship courses/lectures at your medical school *Regular use is defined as reported utilization “often” or “most of the time.”

The students were slightly more satisfied with in-person courses than with online courses, and because of this, the students were pushed toward using board-specific resources. Over three-quarters of the students involved reported that a majority of their learning was from board-specific resources; this includes the students who watched all (or nearly all) of the class lectures. These findings are consistent with the current literature (Figure 3) [13], and although the students choose different board-specific resources, they are choosing the said resources over their school resources, despite the added cost of purchasing outside resources. The implications of such findings are extensive and should cause medical schools to evaluate both the content they deliver and the methods through which they deliver it. As such, curriculum developers may need to seriously question the efficacy of lectures and how they may be replaced, which could be done in-house or potentially by third-party creators.

This study highlights the notion that the current system for medical education is less effective than it is intended to be and that COVID-19 brought such disparities to light. United States medical students are paying an average of $47,600 per year in tuition alone to go to medical school only to learn the material from independent, online resources for a fraction of the cost [14]. In addition, the COVID-19 pandemic revealed that students do not need to be in-person for the entirety of their medical education. This is especially true for traditional learning methods, such as lectures and small group activities [15]. While many students disliked the feelings of isolation and reported decreased motivation with online learning, others preferred the efficiency with which they were able to learn and preferred the virtual setting as a result. This could eventually lead to the creation of hybrid medical schools, where the only in-person activities are clinical.

These trends are subject to change, especially with USMLE Step 1 moving to pass/fail. It remains to be seen how this will affect students’ resource preferences, especially in their pre-clerkship years, but over 50% of the students believe that they would spend less money on board-specific test preparation resources, such as online videos and question banks. Educators also worry that students will spend less time studying, replacing the time they used to vigorously study for the USMLE Step 1 with time spent on other activities important to their residency applications, such as research and volunteering [16]. The feared outcome here would be students struggling during rotations because of a lack of fundamental medical knowledge. In addition, many anticipate that the USMLE Step 2 examination will take the place of the USMLE Step 1 examination, in that the USMLE Step 2 examination will likely be used to distinguish students in terms of residency application [17]. The result will likely be similar to the current Step 1 situation - students will dedicate great amounts of time and money to board-specific resources to improve their scores and thus their chances of matching into their residency of choice.

In an attempt to improve the curriculum with the engagement that only schools are able to provide, we offer a potential suggestion: utilizing a flipped classroom model where students independently learn via the resource of their choosing and subsequently gather as a class in small groups for team-based learning. This format could revolve around the school’s curriculum developers creating real-world cases that students can work through along with board-style questions where students can solidify their understanding in the team-based setting. Hew and Lo found that using a flipped classroom approach in medical education resulted in significantly improved student learning when compared with traditional teaching methods [18]. Although flipped classrooms can be difficult to implement due to the drastic shift in pedagogy away from tradition, the potential benefit to medical students warrants serious consideration. Additionally, if the school has the capacity, curriculum developers could also create practice clinical simulations for students to further solidify their knowledge by practicing real-life patient encounters revolving around the material they have just learned. In this way, students can learn the material, evaluate their knowledge with board-style questions and real-world cases, teach their peers in the classroom setting, and practice their knowledge in the clinical setting.

Future studies may want to examine the prevalence of board-specific resources after USMLE Step 1 changes to a pass/fail format, which could possibly decrease the likelihood that students will spend extra money on resources that they may not see as essential, at least until the USMLE Step 2 examination. Additionally, while students at our school reported adequate access to external resources, this may not be the case in other medical schools. Furthermore, it would be interesting to see how board-specific resource use correlates with USMLE Step 1 scores and third-year clerkship grades because if students who attended fewer lectures received higher grades, this should further incentivize schools to strongly reconsider the content they deliver.

One limitation is that the courses studied here involved different subjects and different course directors, who may organize the course in slightly different ways. As such, the ability to exactly compare the in-person setting with the online setting may be hindered. In addition, the class under study had only 64 students, so the capacity to generalize on a national and international scale is limited.

## Conclusions

The onset of COVID-19 required medical schools and medical students to adapt their approach to medical education. This study compared first-year medical students’ perceptions of an online curriculum, focusing on the students’ preferred resources before and after the transition to virtual courses, and found that pre-clerkship medical students preferred some aspects of the in-person setting and some aspects of the virtual setting. In terms of medical education resources, before and after the transition, students were less satisfied with traditional curricular resources and more likely to choose external, board-specific resources with hopes of building strong residency applications, and these preferences were heightened in the online format.
